# Application of modified small bladder patch-to-bladder double-layer sutures to improve renal transplantation in mice

**DOI:** 10.1007/s10353-016-0391-7

**Published:** 2016-06-09

**Authors:** Chen Wenwei, Yang Yirong, Katarzyna M. Stevens, Michal Heger, Xia Peng

**Affiliations:** 1 Department of Transplantation, The First Affiliated Hospital of Wenzhou Medical University, Shangcai Village, Ouhai District, 325000 Wenzhou, Zhejiang P. R. China; 2 Live Cell Imaging Facility, Snyder Institute for Chronic Diseases, University of Calgary, Calgary, Alberta Canada; 3 Department of Experimental Surgery, Academic Medical Center, University of Amsterdam, Amsterdam, The Netherlands

**Keywords:** Experimental model, Mouse, Renal transplantation, Ureteral obstruction, Urinary tract reconstruction

## Abstract

**Background:**

This study aimed to introduce an improved surgical procedure to reduce the incidence of urinary tract complications after renal transplantation in mice using a modified bladder patch-to-bladder anastomosis technique.

**Methods:**

Renal isotransplantation was performed in 28 male C57BL/6 mice. The urinary tract was reconstructed with a ureteral anastomosis between the donor’s small bladder patch and the recipient’s bladder. The bladder patch was secured through a cystotomy in the recipient’s bladder mucosa and seromuscular layers, which were sutured in a double-layer manner. The food intake and survival of mice were recorded for 100 days in addition to monitoring appearance, weight, and symptoms of pain. On post-transplantation day 7, the native kidney in the recipients was removed and the transplanted kidney assessed visually. Urine leakage from the transplanted graft was monitored by assessing the degree of ascites.

**Results:**

The success rate of renal transplantation was 82 % (23 of 28 cases). Arterial thrombosis at the site of anastomosis occurred in 3 cases (11 %) and hemorrhagic shock in 2 cases (7 %). The mean ± SD time of the operation in recipients was 81 ± 5 min. No complications were noted in the successfully transplanted animals.

**Conclusions:**

The modified procedure of a small bladder patch-to-bladder with double-layer suturing minimizes complications after renal transplantation in mice while requiring the same operating time as other approaches such as ureter to bladder anastomosis, which are associated with more complications.

## Introduction

The mouse kidney transplantation model [1–3] is an important translational research tool because it mimics the clinical features of kidney transplantation and allows the controlled investigation of pharmacological interventions [4, 5] as well as (patho)physiological [6, 7], biological [8–13], and biochemical [14, 15] processes. Moreover, the advent of genetically modified mice enabled the investigation of immunological mechanisms that play a role in transplantation outcome and graft rejection [16, 17].

Although very useful for kidney transplantation research, the murine renal transplantation model is currently performed in only a few transplantation centers in the world, due to the very long learning curve and the technical complexity of the procedure, which results in high mortality rates [18]. In addition to the vascular anastomosis following the transplantation procedure [18], the urinary tract reconstruction (UTR) constitutes a significant technical challenge. A flawed UTR can cause urinary tract complications and impair graft quality, function, and viability. Minimizing the complexity of the UTR is therefore essential to reduce procedure-related complications, lower the mortality rate, and produce translatable research results [18, 19].

In 1995, Zhang et al. [3] standardized the kidney transplantation procedure in mice, which was further fine-tuned by Martins in 2006 [20] by the application of a bladder patch-to-cystotomy to prevent vesicoureteral reflux. But the technique is time-consuming and complicated. In this technical paper, we simplified the procedure by using a small bladder patch-to-bladder double-layer suture approach to reconstruct the urinary tract, which shortened the operation time and minimized urinary tract complications.

## Materials and methods

### Animals

The animal experiments were approved by the animal ethics committee of the First Affiliated Hospital of the Wenzhou Medical University (protocol # WYDW2013-0002) and animals received care in compliance with the NIH *Guide for the Care and Use of Laboratory Animals* (eighth edition, 2011). Male inbred C57BL/6 mice were housed in a controlled environment with 12-h dark and light cycles and ad libitum access to standard chow and water. Mice (n = 56) weighing 20–30 g were randomly selected as donors and recipients.

### Transplantation procedure

Prior to surgery, 0.5–1.0 mL of 5 % glucose was fed to the recipient mice to maintain the filling of the bladder during the procedure. Pentobarbital sodium (60 mg/kg) was given by intraperitoneal injection for induction and maintenance anesthesia [21]. All procedures were performed using a surgical stereomicroscope (ALLEGRA900 Möller–Wedel, Germany) with external white light illumination at 5–20 × magnification and standard microsurgical instruments.

**Fig. 1:**
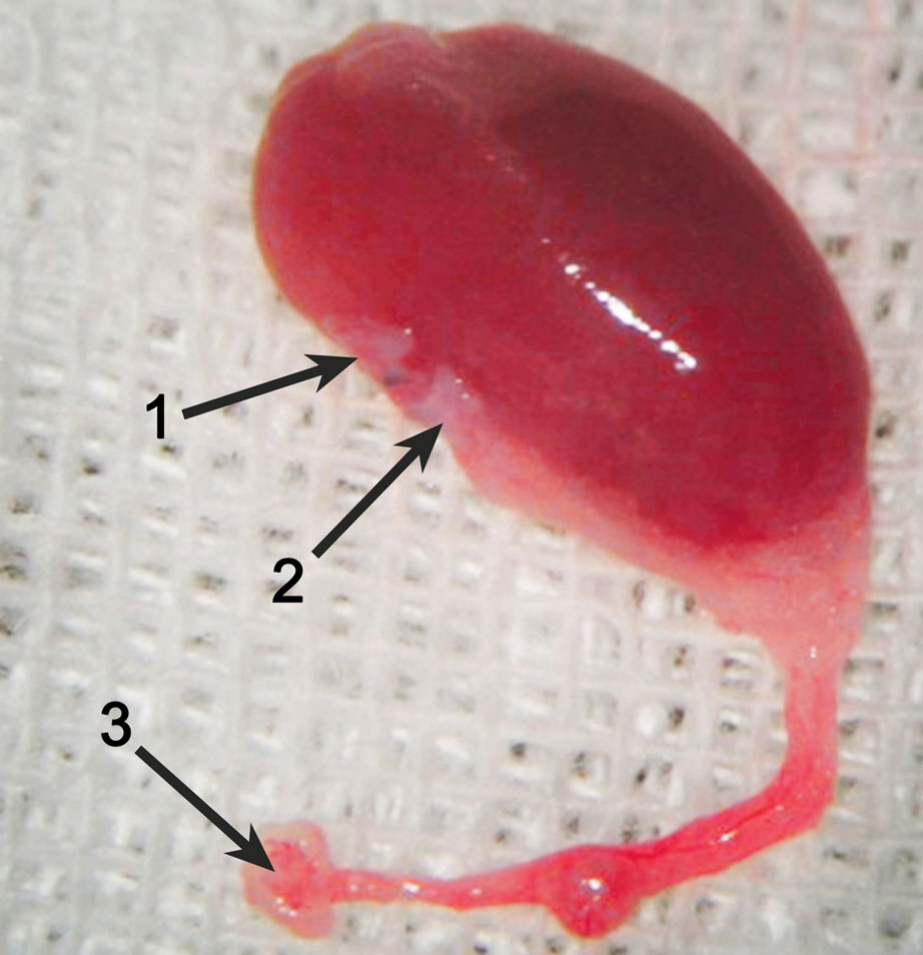
The kidney graft after perfusion. (*1* Left renal vein with the vena cava valve, *2* left renal artery with abdominal aortic Carrel patch, *3* bladder patch)

Kidney harvesting was performed as described by Martins [20]. Briefly, the periureteral vessels and fat were preserved while the ureter was cut with a bladder patch of 1.0–2.0 mm in diameter. The kidney graft was resected by cutting the ligated left renal vein and left renal artery with a Carrel patch in the inferior vena cava and aorta, respectively (Fig. 1).

**Fig. 2:**
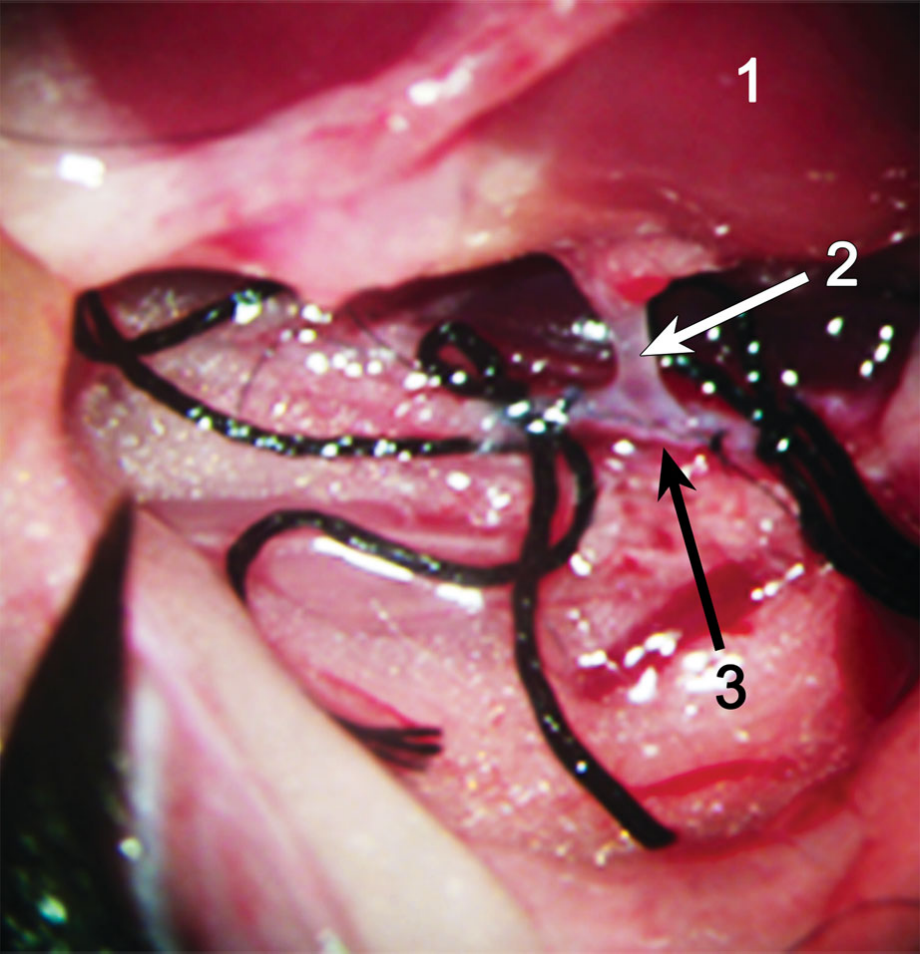
The anastomosis between the recipient abdominal aorta and the aorta Carrel patch of the donor kidney. (*1* Donor kidney, *2* left renal artery, *3* recipient’s abdominal aorta)

**Fig. 3:**
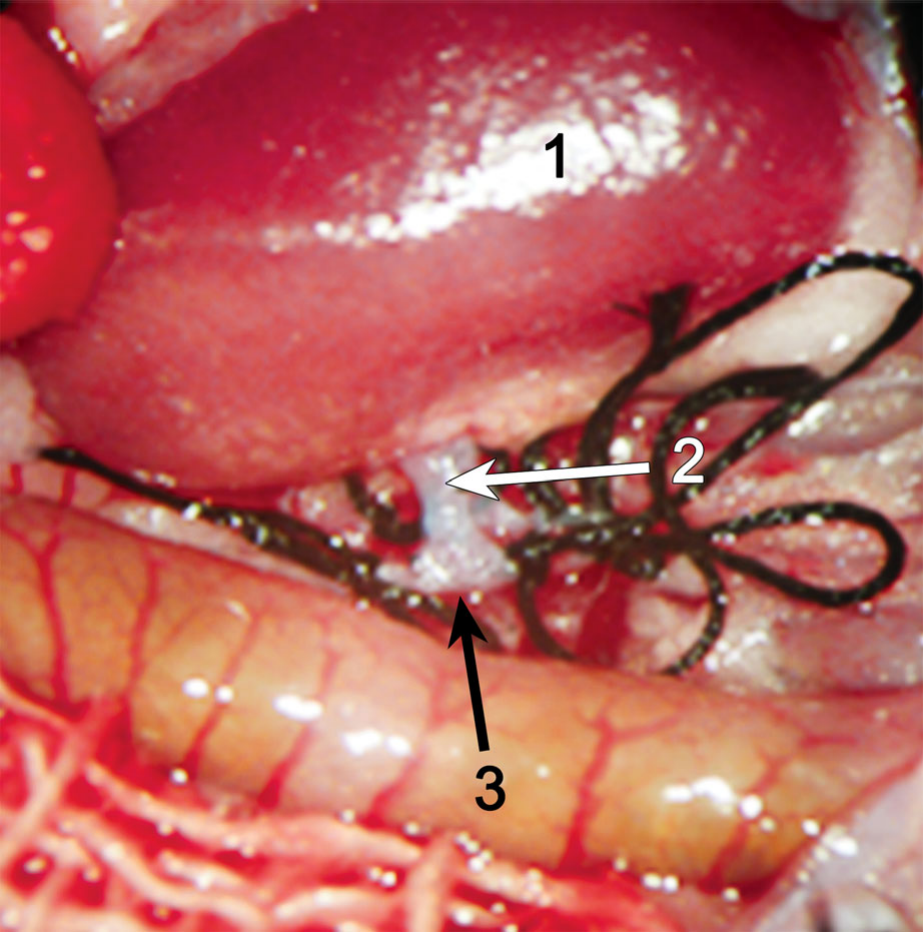
The anastomosis between the recipient inferior vena cava patch and the inferior vena cava of the donor kidney. (*1* Donor kidney, *2* left renal vein, *3* recipient’s inferior vena cava)

**Fig. 4:**
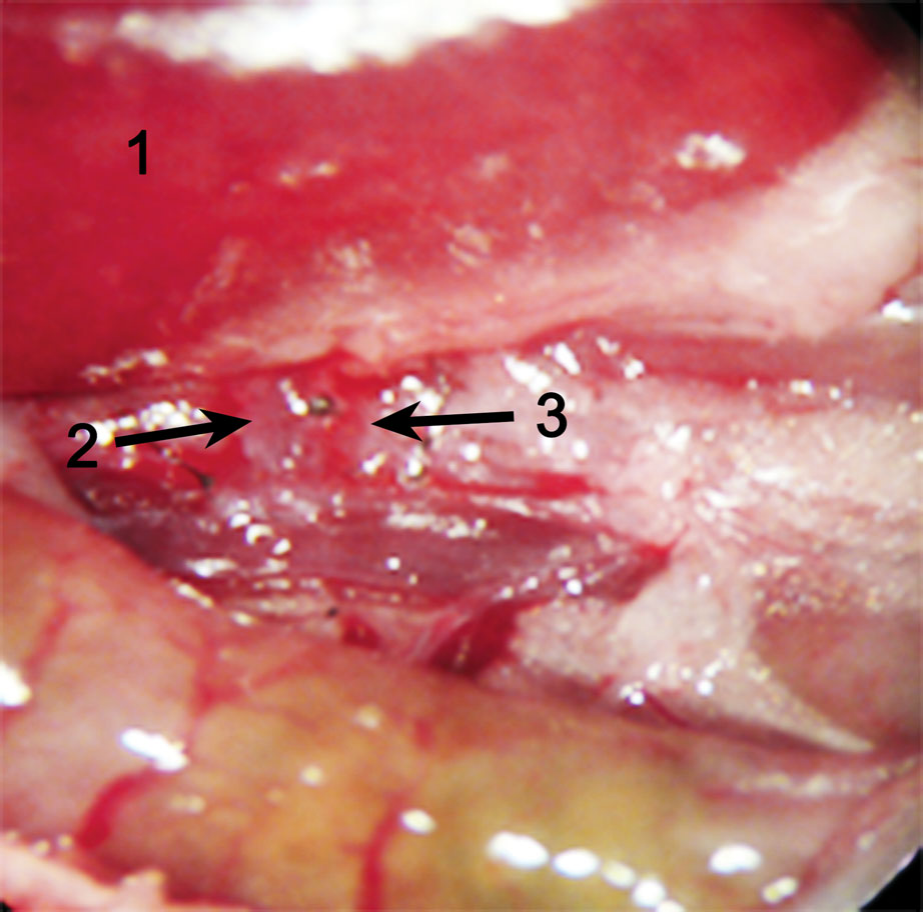
Restored perfusion to the kidney following transplantation. (*1* Donor kidney, *2* left renal vein, *3* left renal artery)

For the kidney implant and UTR in the recipients, the anastomosis between the recipient abdominal aorta and the aorta Carrel patch of the donor kidney was made in an end-to-side manner (Fig. 2). A similar anastomosis was made between the recipient inferior vena cava patch and the inferior vena cava of the donor kidney (Fig. 3). Then, the ligations were loosened, and perfusion to the kidney was reinstated (Fig. 4). The UTR in the recipient mice was achieved by the anastomosis between the donor’s small bladder patch and the recipient’s bladder.

**Fig. 5:**
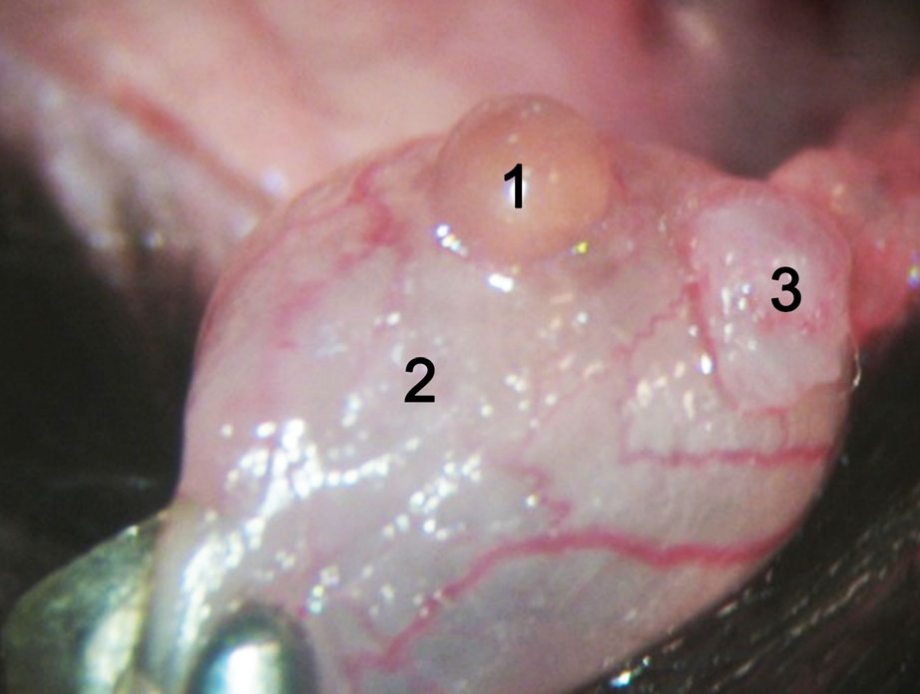
A small incision in an avascular zone on the surface of the recipient’s bladder. (*1* Raised mucosal layer of the bladder, *2* recipient’s bladder, *3* graft small bladder patch)

**Fig. 6:**
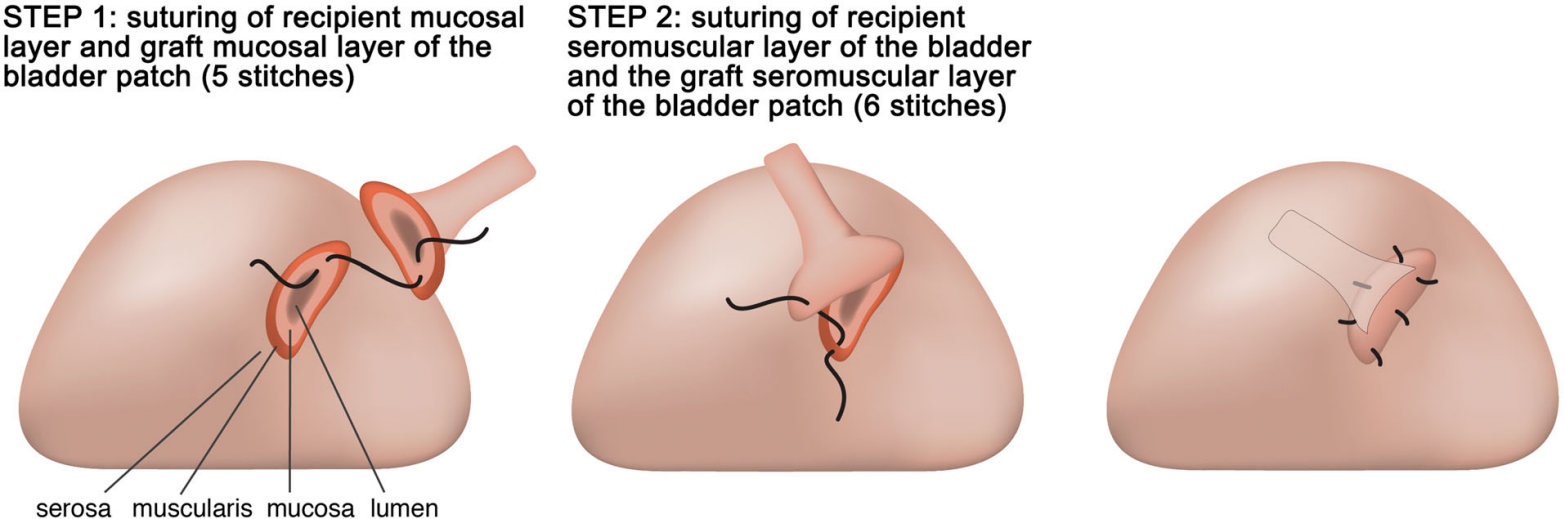
Schematic drawing of the ureterovesical anastomosis steps

**Fig. 7:**
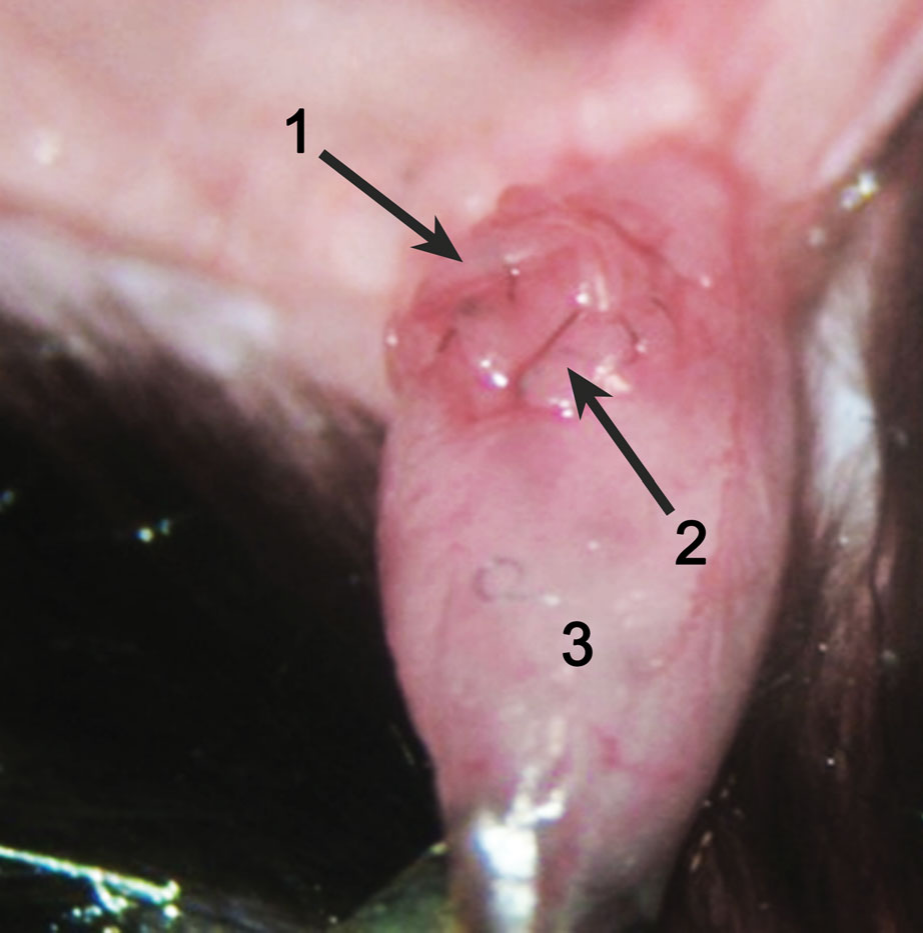
The small bladder patch and the recipient ureter was secured to the recipient bladder wall by continuous suturing to the seromuscular layer (step 2, Fig. 6). (*1* Graft latter part of the ureter, *2* graft small bladder patch, *3* recipient’s bladder)

**Fig. 8:**
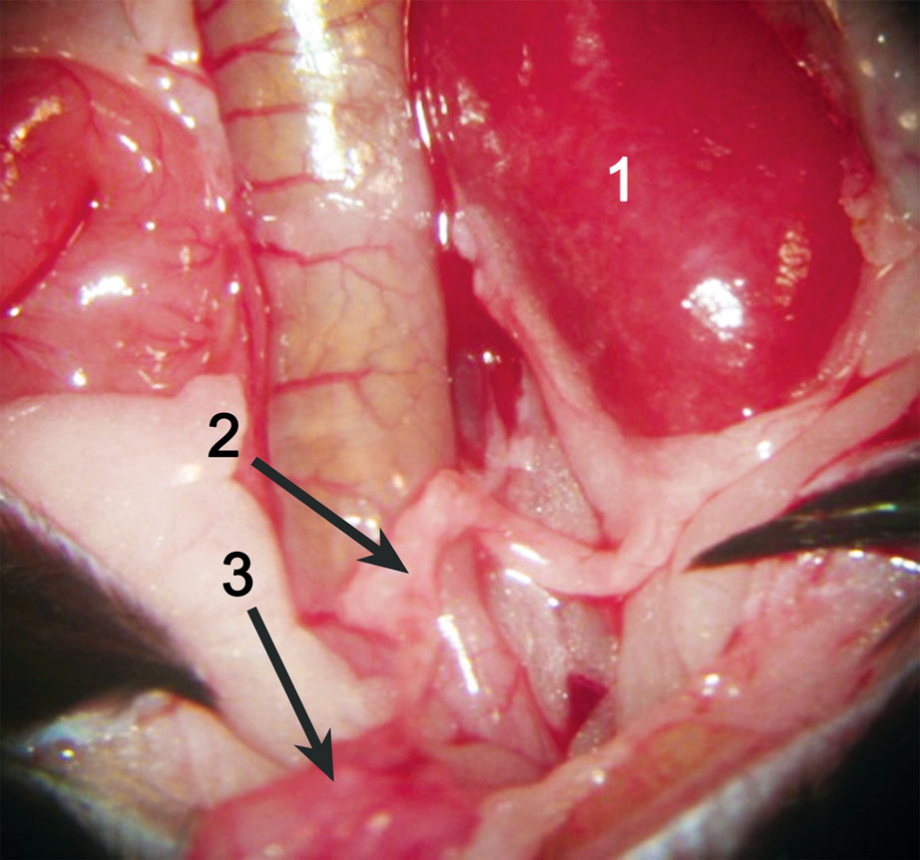
The implanted kidney after transplantation. The kidney is red in color and the ureter is fed by blood supply. (*1* Transplanted kidney, *2* ureter native to the transplanted kidney, *3* small bladder patch and the recipient’s bladder)

The bladder anastomosis was then carried out by making a small incision (1.5–2.0 mm) in the seromuscular layer of the recipient bladder in an avascular zone of the posteriolateral wall of the bladder without perturbing the mucosal layer. A little pressure was then applied to the bladder to separate the mucosal and the seromuscular layers, and consequently the mucosal layer of the bladder protruded outward (Fig. 5). Subsequently, a small incision was made in the swollen mucosal layer. Then the separated recipient mucosal layer and the graft mucosal layer of the bladder patch were sutured together with a series of 5 stitches using 10-0 silk sutures. Finally, the recipient seromuscular layer of the bladder and the graft seromuscular layer of the bladder patch were sutured together with a series of 6 stitches using 10-0 silk sutures (Figs. 6 and 7). A successful renal transplantation was characterized by an implanted kidney that was red in color, indicating sufficient blood supply to the ureter, and a bladder patch with a pink flesh color (Fig. 8). During the operation, a heating lamp was used to maintain the animal’s body temperature near euthermia.

### Postoperative care and observation

After implantation, recipient mice were injected subcutaneously with 0.5 mL sterile saline. The mice were placed in a 37 °C incubator until they recovered from the anesthesia. Once awake, the food intake and survival of mice were recorded for 100 days in addition to monitoring appearance, weight, symptoms of pain (i. e., behavioral patterns), and cage bedding (for hematuria) in accordance with [22, 23]. On post-transplantation day 7, the autologous kidney in the recipients was removed as described in Section 2.2 and the viability of the transplanted kidney was assessed visually (e. g., necrotic patches in the cortex and at the anastomotic site). Urine leakage from the transplanted graft was monitored by assessing the degree of ascites.

## Results

Twenty-eight mice received syngeneic renal transplants by UTR using a modified small bladder patch-to-bladder approach of anastomosis. During the surgical procedure, the average time spent on kidney harvesting was 35 min. The mean ± SD time of the operation in recipient mice was 81 ± 5 min. The average renal warm ischemia time, as defined by Ge and Gong [19], was 25 min, corresponding to mild ischemic injury time [24]. As a result, the surgical success rate was 82 % (23 cases). The 5 failed cases were due to arterial thrombosis at the site of anastomosis (3 cases, 11 %) and hemorrhagic shock (2 cases, 7 %) during the transplantation procedure.

**Fig. 9:**
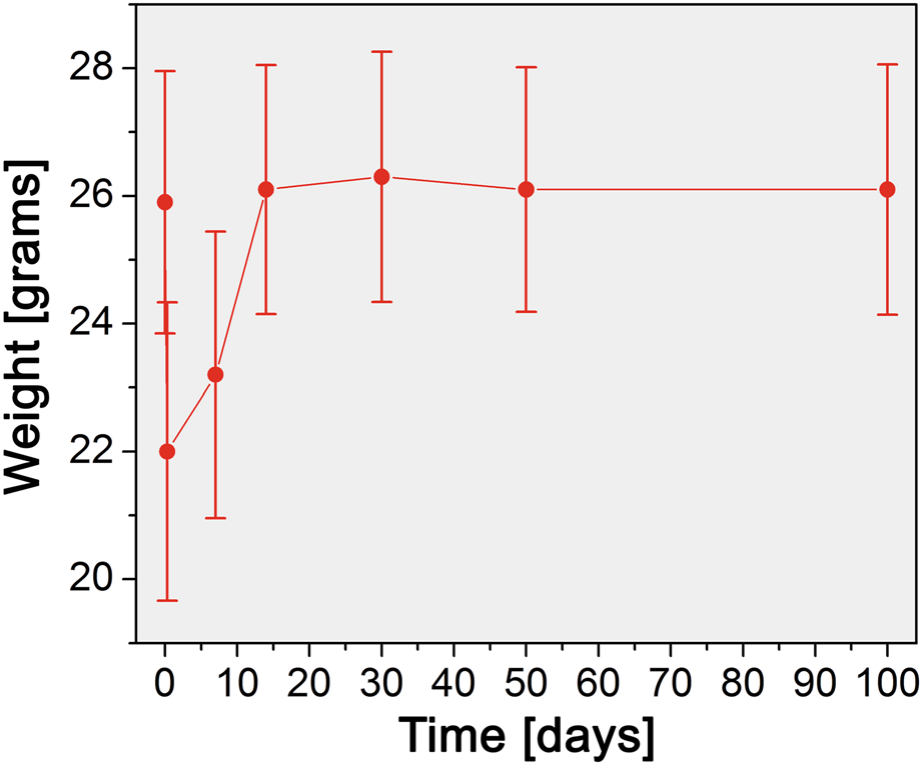
Animal weights recorded before transplantation (0 days) and at 8 h and 7, 14, 30, 50, and 100 days after transplantation. Data represent mean ± SD of N = 23 animals

In the remaining 23 cases in which the transplantation procedure was successful, the mice ate approximately 8 h after surgery, and no apparent complications or premature death (< 7 days) occurred. The animals lost weight directly after the transplantation (3.9 grams on average), which was restored to pre-surgery levels within 14 days and remained constant during the remainder of the experiment (Fig. 9). The recipient mice survived for 100 days, during which no transplantation-related symptoms such as hydronephrosis (inflammation, hematuria, or signs of kidney failure) were observed.

## Discussion

Complications associated with vascular anastomosis during renal transplantation in mice include anastomotic stricture, bleeding, and thrombosis [18]. Urinary tract complications include urine leakage, ureter constriction or obstruction, necrosis, and hematuria [19]. Furthermore, complications arise due to the length of surgery, such as hemorrhagic shock and paraplegia. As surgeons gradually improve the vascular anastomosis technique, the operation time gradually shortens [18]. Consequently, the corresponding complications decrease [20], albeit these still remain relatively high in mice [18]. Urinary tract complications are notable as they occur in 7 % of cases, which are mainly related to the method of reconstruction and complications associated with the anastomosis [3, 25].

Currently, common methods used for UTR during renal transplantation in mouse models involve ureter-to-bladder anastomosis and bladder patch-to-bladder anastomosis approaches without double-layer suturing [3]. These conventional bladder patch-to-bladder anastomosis approaches use interrupted and unilateral suturing methods. Due to the thickness of the bladder wall and a “non-filling” state of the recipient’s bladder, the required area of the bladder patch can be large and may lead to an increased probability of bladder patch ischemia, in turn causing bladder patch necrosis, urine leakage, urinary tract obstruction, and other urinary system complications [19]. In addition, the reconstruction method of the ureter-to-bladder anastomosis or bladder patch-to-bladder interrupted suture does not entail the use of internal drainage tubes or catheters. Urine leakage is, therefore, more likely to occur after operation. Often, urine leakage is difficult to observe after the operation and may be overlooked. Urine leakage may consequently lead to infections or sclerosis at locations of anastomoses and the distal ureter, increasing the incidence of ureter obstruction.

In preliminary experiments we used ureter-to-bladder anastomosis for UTR as described by Wang [25]. Ureteral obstruction and graft pyonephrosis occurred at a high incidence (data not shown). A method of bladder patch suturing to an incision on the bladder was reported for the reconstruction of the urinary tract by Martins [20]; thus was associated with a reduction in urine leakage and urinary tract obstruction. However, the technique is time consuming and difficult to perform. In order to overcome the above-mentioned drawbacks of the UTR, our surgical procedure was designed to carefully protect the ureter blood supply during a small bladder patch anastomosis, followed by continuous suturing of the mucosal and seromuscular layer of the bladder. As a result, 23 transplanted mice (82 %) did not exhibit signs of transplantation-related complications and survived for 100 days.

Compared to the technique by Martins [20], we improved the small bladder patch-to-bladder suture approach by simplifying the steps. As a result, our method is equally time consuming (81 min in our study for 28 mice versus ~ 83 min in Martins’ study after 30 animals [18]) but with less complications and fatalities. Martins reported 20 % vascular occlusion, 30 % arterial thrombosis, 10 % hemorrhage, 30 % intraoperative failure, 20 % death within 7 days, and 8 % survival after 90 days [18]. Our technique was associated with 11 % arterial thrombosis, 7 % hemorrhagic shock, no complications within 7 days after the transplantation, and 82 % survival rate after 100 days. Moreover, we used a smaller bladder patch than what was required by Zhang et al. [3] for their single-layer sutures approach to prevent leakage and necrosis. The smaller bladder patch-to-bladder anastomosis is associated with reduced risk of post-operative ureteral obstructions [20] compared to ureter-to-bladder anastomosis [25], as is reflected by the acceptable statistics of our results. In the case of ureter-to-bladder anastomosis, the bladder wall may press on the ureter, leading to ureter stricture after kidney transplantation (2–7 % of cases) [26–28].

Compared to the conventional approach of the bladder patch-to-bladder anastomosis, the modified small bladder patch-to-bladder double-layer suture approach has the following practical advantages: 1) the use of a small patch can reduce ischemia and necrosis of the bladder patch; 2) a small incision is made in the bladder, thus a continuous double layer of sutures is sufficient to avoid urine leakage, and the secured bladder patch can effectively prevent reflux of the ureter [3, 19]; and 3) the raised bladder mucosal layer, which is separated from the seromuscular layer, provides a clear view and allows for easy bladder dissection.

## Conclusion

The modified procedure of a small bladder patch-to-bladder with double-layer suturing minimizes the rate and extent of complications after renal transplantation in mice while requiring the same operating time as other approaches such as ureter-to-bladder anastomosis, which are associated with more severe complications occurring at a higher rate.
